# Strigolactones affect the yield of Tartary buckwheat by regulating endogenous hormone levels

**DOI:** 10.1186/s12870-024-05029-0

**Published:** 2024-04-24

**Authors:** Zhuolei Tang, Xiaoyan Huang, Kaifeng Huang

**Affiliations:** School of Life Science, Guizhou Normal University, Guiyang, 550001 China

**Keywords:** Tartary buckwheat, Strigolactones, Endogenous phytohormones, Branches, Yield

## Abstract

**Background:**

As a newly class of endogenous phytohormones, strigolactones (SLs) regulate crop growth and yield formation by interacting with other hormones. However, the physiological mechanism of SLs affect the yield by regulating the balance of endogenous hormones of Tartary buckwheat is still unclear.

**Results:**

In this study, a 2-year field experiment was conducted on Tartary buckwheat (Jinqiao 2) to study the effects of different concentrations (0, 10, and 20 µmol/L) of artificial synthetic analogs of SLs (rac-GR24) and inhibitor of SL synthesis (Tis-108) on the growth, endogenous-hormone content, and yield of Tartary buckwheat. The main-stem branch number, grain number per plant, grain weight per plant, and yield of Tartary buckwheat continuously decreased with increased rac-GR24 concentration, whereas the main-stem diameter and plant height initially increased and then decreased. Rac-GR24 treatment significantly increased the content of SLs and abscisic acid (ABA) in grains, and it decreased the content of Zeatin (Z) + Zeatin nucleoside (ZR). Conversely, Tis-108 treatment decreased the content of SLs and ABA but increased the content of Z + ZR. Results of correlation analysis showed that the content of ABA and SLs, the ratio of SLs/(Z + ZR), SLs/ABA, and ABA/(Z + ZR) were significantly negatively correlated with the yield of Tartary buckwheat, and that Z + ZR content was significantly positively correlated with the yield. Regression analysis further showed that ABA/ (Z + ZR) can explain 58.4% of the variation in yield.

**Conclusions:**

In summary, by adjusting the level of endogenous SLs in Tartary buckwheat, the balance of endogenous hormones in grains can be changed, thereby exerting the effect on yield. The results can provide a new agronomic method for the high-yield cultivation of Tartary buckwheat.

**Supplementary Information:**

The online version contains supplementary material available at 10.1186/s12870-024-05029-0.

## Background

Tartary buckwheat (*Fagopyrum tataricum* (L.) Gaertn.) is one of the main cultivated species of buckwheat (*Fagopyrum esculentum* Moench). It is a false cereal crop with chemical composition and practical use similar to those of rice, wheat, and other grains [1]. Tartary buckwheat has strong ecological adaptability and can be extensively planted in many parts of the world [2]. Tartary buckwheat grains contain various bioactive components such as flavonoids and polyphenols, which positively affect the prevention and adjuvant treatment of diseases, such as cardiovascular, gallstones, and diabetes [3]. At present, Tartary buckwheat-based products including tea, bread, and flour attract increased attention due to their outstanding nutritional and health values [4]. However, due to the low and unstable yield of Tartary buckwheat in production, its planting and promotion are greatly limited. Therefore, the realization of high-yield cultivation is a scientific problem to be solved in the process of promoting the industrialization of Tartary buckwheat [5].

Grain weight per plant and grain number per plant are important components of crop yield [6, 7]. In most cases, there are positively correlated with crop yield [8]. Mousavi and Nagy (2021) found that the yield of maize is positively correlated with grain weight per plant and 1000-grain weight [9]. Pirzado et al. (2021) found a significant positive correlation between wheat yield and grain number per panicle [10]. High-yield Tartary buckwheat varieties generally have suitable agronomic characteristics such as plant height, number of main-stem branches, and number of internodes. Such plant types have advantages in terms of group competitive growth and being difficult lodging, which are conducive to the growth, seed setting, and yield of Tartary buckwheat [11]. Kolarić et al. (2021) found that the yield of Tartary buckwheat has a significant positive correlation with plant height, branch number of main stem, leaf number [12]. These studies show that yield can be affected by changing the plant type of Tartary buckwheat.

Endogenous hormones such as auxin (IAA), cytokinin (CTK), and abscisic acid (ABA) play important roles in regulating crop growth. They jointly participate in the growth and development of plants [13], wherein ABA and CTK play important roles in crop yield [14]. ABA has the function of coordinating multiple major growth and development processes, which can promote the elongation of plant main root and regulate the development of grain embryo [15, 16]. CTK is a kind of plant hormone that can enhance cell division, induce bud formation, and promote its growth. It includes natural CTKs such as zeatin (Z) and zeatin riboside (ZR) in plants, as well as synthetic CTKs such as kinetin and 6-benzylaminopurine [17]. Cai et al. (2014) showed that the exogenous application of IAA and ABA can affect the content of IAA and Z in wheat and the ratio of ABA/Z, thereby regulating tillering, promoting nutrient accumulation, and increasing the yield of wheat [18]. The stable growth of plants requires a dynamic balance among endogenous hormones. By applying exogenous hormones or hormone-synthesis inhibitors, the level of endogenous hormones in plants can be changed. Thus, different parts of plants can grow in various ways, and the growth direction and development process of plants can then be regulated [19].

Strigolactones (SLs) are a newly discovered class of plant hormones that regulate the growth of plant roots, branches, and hypocotyls [20, 21]. Yamada et al. (2019) believed that SLs can regulate rice endosperm development and increase yield [19]. Ma et al. (2020) used rac-GR24 (artificial synthetic analogs of SLs) to promote lateral root growth, thereby increasing the yield of rapeseed [22]. SLs also interact with ABA and CTK. Toh et al. (2012) found that SLs can alleviate heat stress by regulating ABA content in plants [23]. Yoneyama et al. (2020) found that the application of CTK can inhibit the level of SLs in the root tissue of rice plants [24]. Based on the results of the above studies, the current study hypothesized that SLs may affect the yield by regulating the balance of endogenous hormones of Tartary buckwheat. However, studies relevant to this hypothesis are lacking. In the present study, the effects of rac-GR24 and inhibitor of SL synthesis (Tis-108) on the branching, endogenous hormone levels, and yield of Tartary buckwheat were studied. The major objectives were as follows: (1) evaluate the effects of rac-GR24 and Tis-108 on the growth, (2) analyze the effects of rac-GR24 and Tis-108 on the content of endogenous hormones (ABA and Z + ZR), and (3) clarify the regulation of rac-GR24 and Tis-108 on the yield of Tartary buckwheat. This study has great significance for revealing the physiological mechanism of SLs regulating the yield formation of Tartary buckwheat. It also provides a new agronomic method for the high-yield cultivation of Tartary buckwheat.

## Materials and methods

### Plant materials and growth

Tartary buckwheat “cv Jinqiao 2” (JQ2, popular and widely cultivated cultivar of Tartary buckwheat in Guizhou Province, China) was provided by School of Life Science of Guizhou Normal University, China. SLs (rac-GR24, a synthetic analog of SLs, C_17_H_14_O_5_, purity > 98%) and endogenous SL synthesis inhibitor (Tis108, C_20_H_21_N_3_O2, purity > 98%) were purchased from Beijing Coolaber Technology Co., Ltd., Beijing, China (http://www.coolaber.com).

The experiment was conducted during the Tartary buckwheat growing season (August–November) from 2019 to 2020 at Xiaba’s Cultivation Experiment Station of Guizhou Normal University, Guiyang City, Guizhou Province, China (1250 m, 106.94°E, 26.73°N). The area had a subtropical plateau monsoon humid climate. The annual average temperature was 14.95℃, the annual average precipitation was 1319.05 mm. The soil used was yellow loam with 20.01 mg/kg available phosphorus, 24.68 mg/kg available potassium, 8.23 mg/kg ammonium nitrogen, 28.67 mg/kg nitrate nitrogen, and 27.95‰ organic matter. Soil nutrient contents were determined using a multichannel intelligent soil nutrient meter (OK − Q3, China).

The experiment was performed using a single-factor randomized block design with three replicates. The area for each test plot had dimensions of 10 m^2^ (2 m×5 m). Seeds were sown in the plot on 29 August 2019 and 29 August 2020. The row spacing and seeding amounts were 0.33 m and 3.75 g/m^2^, respectively, and approximately 90–100 reserved plants were available for each square meter. In accordance with the local optimal dosage, 600 kg/hm^2^ compound fertilizer was applied as base fertilizer (N: P: K = 15: 15: 15) in each plot at one time [25]. No fertilizer was applied throughout the entire growth period. In accordance with a previous study, the spraying concentration of rac-GR24 and Tis-108 are 10 and 20µmol/L, which are denoted as G_10_, G_20_, T_10_, and T_20_, respectively. The same amount of water was sprayed as the control treatment (CK, 0 µmol/L), and each treatment was planted in one plot. Rac-GR24 and Tis-108 were sprayed at the early budding stage of Tartary buckwheat (October 12, 2019 and October 13, 2020). The entire Tartary buckwheat plant was sprayed with an appropriate amount to form water droplets on the leaves. Spraying was performed continuously for 4 days to ensure effectiveness. Grains were harvested on November 18, 2019 and November 19, 2020 when 75% of the grains had matured. Artificial irrigation was conducted according to the principle of “extreme drought and thoroughly irrigated” and other field-management and pest-control strategies were consistent with those of local high-yield cultivation [2]. The monthly average temperatures from August to November in 2019 and 2020 were 18.1 °C and 17.0 °C, and the monthly average sunshine hours were 139.1 h and 118.2 h, respectively. The weather station is about 4.2 km away from the trial site.

### Sample preparation

Plants with uniform growth and without diseases and insect pests were selected from the plots of each treatment. Five days after rac-GR24 and Tis-108 spraying, about 2000 flowers (per plot, located on the top one to three nodes of the main stem) that boomed on the same day were marked on the calyx with a brush dipped in black ink. After 5 days, marked flowers were sampled for the first time and every 5 days until maturation [7]. In each plot, 50 labeled grains were collected every time. These grains were frozen in liquid nitrogen for 30 s and stored in a − 80 °C refrigerator for the determination of endogenous-hormone content.

### Measurement

#### Determination of agronomic traits and yield

Six Tartary buckwheat plants with uniform growth were randomly selected from each plot at mature stage. Plant height and main-stem diameter were measured with a tape and vernier caliper, and the number of main-stem branches was calculated [2]. The number of grains per plant and grain weight per plant was determined using a seed-test analyzer (SC-A, Hangzhou Wanshen Exploration Technology Co., Ltd., China. https://www.wseen.cn). In the middle of each treatment plot, the grains on all Tartary buckwheat plants within the 1 m^2^ area (randomly selected, not sampled during the experimental process, excluding border plants) were used to determine the yield after air drying [5].

#### Determination of endogenous-hormone content

A fresh grain sample (0.1 g) after shelling was added to 2 mL of PBS (pH 7.4), pre-cooled, and ground for use. The contents of SLs, ABA, and Z + ZR were analyzed by enzyme-linked immunosorbent assay. The kit was purchased from Wuhan Purity Biotechnology Co., Ltd., China (http://chundubio.com).

### Statistical analysis

Microsoft Excel 2010 was used for data processing. IBM SPSS 27.0 was used for single-factor difference analysis (ANOVA) and regression analysis. One-way ANOVA was performed, and means were compared by using the least significant difference at the 0.05 probability level. The correlation analysis was analyzed using an online data analysis platform SPSSPRO (https://www.spsspro.com). OriginPro 2024 was used for data mapping. The results of 2019 and 2020 were similar. Accordingly, the data were presented as the average across the two study years, and the data of 2019 and 2020 were deposited as supplementary data.

## Results

### Effects of rac-GR24 and Tis-108 on the agronomic traits and yield of Tartary buckwheat

The main-stem branch number, grain number per plant, and grain weight per plant of Tartary buckwheat decreased continuously with increased rac-GR24 concentration, whereas the main-stem diameter and plant height initially increased and then decreased (Table 1). Compared with the CK treatment, Tis-108 treatment increased the number of main-stem branches, main-stem diameter, plant height, grain number per plant, and grain weight per plant by average of 15.14%, 27.68%, 35.14%, 50.00%, and 69.47%, respectively. Compared with the CK treatment, G_10_ and G_20_ treatments reduced the yield of Tartary buckwheat by 5.53% and 24.12%, respectively, whereas T_10_ and T_20_ treatments increased by 20.60% and 4.52%, respectively.

**Table 1:** Effects of different concentrations of rac-GR24 and Tis-108 on Agronomic characters and yield of Tartary buckwheat

Treatment		Number of branches	Diameter of main stem (mm)	Plant height (cm)	Grain number per plant	Grain weight per plant (g)	Yield (t/ha)
CK	14.33 ± 0.58b	2.98 ± 0.04d	85.92 ± 1.92c	254.00 ± 8.00c	4.57 ± 0.26c	1.99 ± 0.05bc	
G_10_	12.00 ± 1.00c	3.17 ± 0.03c	97.14 ± 1.47b	219.00 ± 13.00d	4.05 ± 0.11d	1.88 ± 0.11c	
G_20_	11.67 ± 0.58c	2.48 ± 0.16e	76.03 ± 4.78c	180.00 ± 13.00e	3.26 ± 0.38e	1.51 ± 0.09d	
T_10_	16.33 ± 1.53a	3.93 ± 0.05a	118.18 ± 8.61a	411.00 ± 27.00a	8.27 ± 0.33a	2.40 ± 0.10a	
T_20_	16.67 ± 0.58a	3.68 ± 0.08b	114.04 ± 7.42a	351.00 ± 20.00b	7.22 ± 0.16b	2.08 ± 0.05b	

Note: Data are presented as mean ± standard error of the mean. Small letter in the same column means significant difference at *p* < 0.05. CK, G_10_, G_20_, T_10_, and T_20_ represent the application of rac-GR24 or Tis108 were 0, 10, and 20µmol/L, respectively

### Effects of rac-GR24 and Tis-108 on endogenous-hormone content in Tartary buckwheat grains

The contents of ABA, Z + ZR and SLs in grains initially increased and then decreased with the advancement of growth period (except that the ABA content in the T_20_ treatment continued to decrease). It reached the maximum 15 or 20 days after marking the flowers (Table 2). Compared with the CK treatment, rac-GR24 treatment increased the content of in grains. The ABA and SLs content of G_20_ treatment was the highest among rac-GR24 treatments. Compared with the CK treatment, rac-GR24 treatment significantly reduced the content of Z + ZR, and the Z + ZR content of G_20_ treatment was the highest. Compared with the CK treatment, Tis-108 treatment reduced the content of ABA and SLs and increased the content of Z + ZR. The contents of ABA and SLs in rac-GR24 treatment were higher than those in Tis-108 treatment, whereas the content of Z + ZR was lower than that in Tis-108 treatment.

**Table 2:** Effects of different concentrations of rac-GR24 and Tis-108 treatments on endogenous hormones in buckwheat

Item	Treatment	Period				
		5d	10d	15d	20d	25d
ABA (ng/ml)	CK	103.06 ± 0.57c	113.40 ± 0.35c	116.90 ± 0.43c	119.43 ± 0.52c	102.29 ± 0.41c
	G_10_	106.41 ± 0.31b	114.79 ± 0.55b	122.69 ± 0.41b	122.50 ± 0.56b	109.33 ± 0.27b
	G_20_	112.87 ± 0.46a	117.09 ± 0.73a	126.67 ± 0.19a	125.76 ± 0.80a	113.93 ± 0.58a
	T_10_	101.91 ± 0.77d	111.79 ± 0.44d	109.43 ± 0.82d	101.86 ± 1.21d	99.07 ± 0.16d
	T_20_	102.86 ± 0.41c	101.38 ± 0.71e	100.42 ± 0.73e	97.40 ± 0.87e	93.62 ± 0.61e
Z + ZR (ng/ml)	CK	9.32 ± 0.03b	10.03 ± 0.04c	11.75 ± 0.07c	11.09 ± 0.10b	10.19 ± 0.14c
	G_10_	8.90 ± 0.02c	9.80 ± 0.03d	10.18 ± 0.04d	10.37 ± 0.11c	9.64 ± 0.50d
	G_20_	8.00 ± 0.10d	8.91 ± 0.02e	10.10 ± 0.07e	9.48 ± 0.10d	8.48 ± 0.11e
	T_10_	9.45 ± 0.17a	10.41 ± 0.13a	14.35 ± 0.06a	11.65 ± 0.05a	10.63 ± 0.06b
	T_20_	9.51 ± 0.02a	10.24 ± 0.10b	13.05 ± 0.04b	11.70 ± 0.09a	11.90 ± 0.08a
SLs (ng/ml)	CK	119.77 ± 0.14b	135.79 ± 0.62c	144.91 ± 0.94c	159.92 ± 0.49b	136.80 ± 0.14c
	G_10_	131.19 ± 0.74a	142.15 ± 1.22b	177.20 ± 0.14b	160.38 ± 0.71b	146.29 ± 0.29b
	G_20_	131.65 ± 0.38a	148.87 ± 0.43a	188.37 ± 0.25a	161.39 ± 0.38a	161.85 ± 0.43a
	T_10_	119.03 ± 0.99c	134.41 ± 0.38d	135.98 ± 0.51d	150.77 ± 0.43c	129.81 ± 1.13d
	T_20_	104.02 ± 0.14d	127.50 ± 0.29e	136.16 ± 0.25d	141.50 ± 0.14d	126.68 ± 0.94e

Note: Data are presented as mean ± standard error of the mean. Small letter in the same column means significant difference at *p* < 0.05. CK, G_10_, G_20_, T_10_, and T_20_ represent the application of rac-GR24 or Tis108 were 0, 10, and 20µmol/L, respectively

### Effects of rac-GR24 and Tis-108 on the ratio of endogenous hormones in Tartary buckwheat grains

The ratios of SLs/ABA, SLs/(Z + ZR), and ABA/(Z + ZR) increased continuously with increased rac-GR24 concentration, and those ratios of G_20_ treatment were significantly higher than those of the other treatments (Table 3). Compared with the CK treatment, the ratios of SLs/ (Z + ZR) and ABA/(Z + ZR) decreased significantly under Tis-108 treatment. The ratios of SLs/ABA, SLs/ (Z + ZR), and ABA/(Z + ZR) in rac-GR24 treatment were higher than those in the Tis-108 treatment.

**Table 3:** Effect of different concentrations of rac-GR24 and Tis-108 treatments on the ratio of endogenous hormones in buckwheat

Treatment	SLs/ABA	SLS/(Z + ZR)	ABA/(Z + ZR)
CK	1.34 ± 0.01b	13.42 ± 0.19c	10.04 ± 0.10c
G_10_	1.34 ± 0.00b	15.22 ± 0.82b	11.37 ± 0.60b
G_20_	1.42 ± 0.01a	19.09 ± 0.27a	13.44 ± 0.11a
T_10_	1.31 ± 0.01c	12.21 ± 0.11d	9.58 ± 0.06d
T_20_	1.35 ± 0.02b	10.65 ± 0.10e	7.87 ± 0.03e

Note: Data are presented as mean ± standard error of the mean. Small letter in the same column means significant difference at *p* < 0.05. CK, G_10_, G_20_, T_10_, and T_20_ represent the application of rac-GR24 or Tis108 were 0, 10, and 20µmolL, respectively

### Correlation analysis

**Fig. 1:**
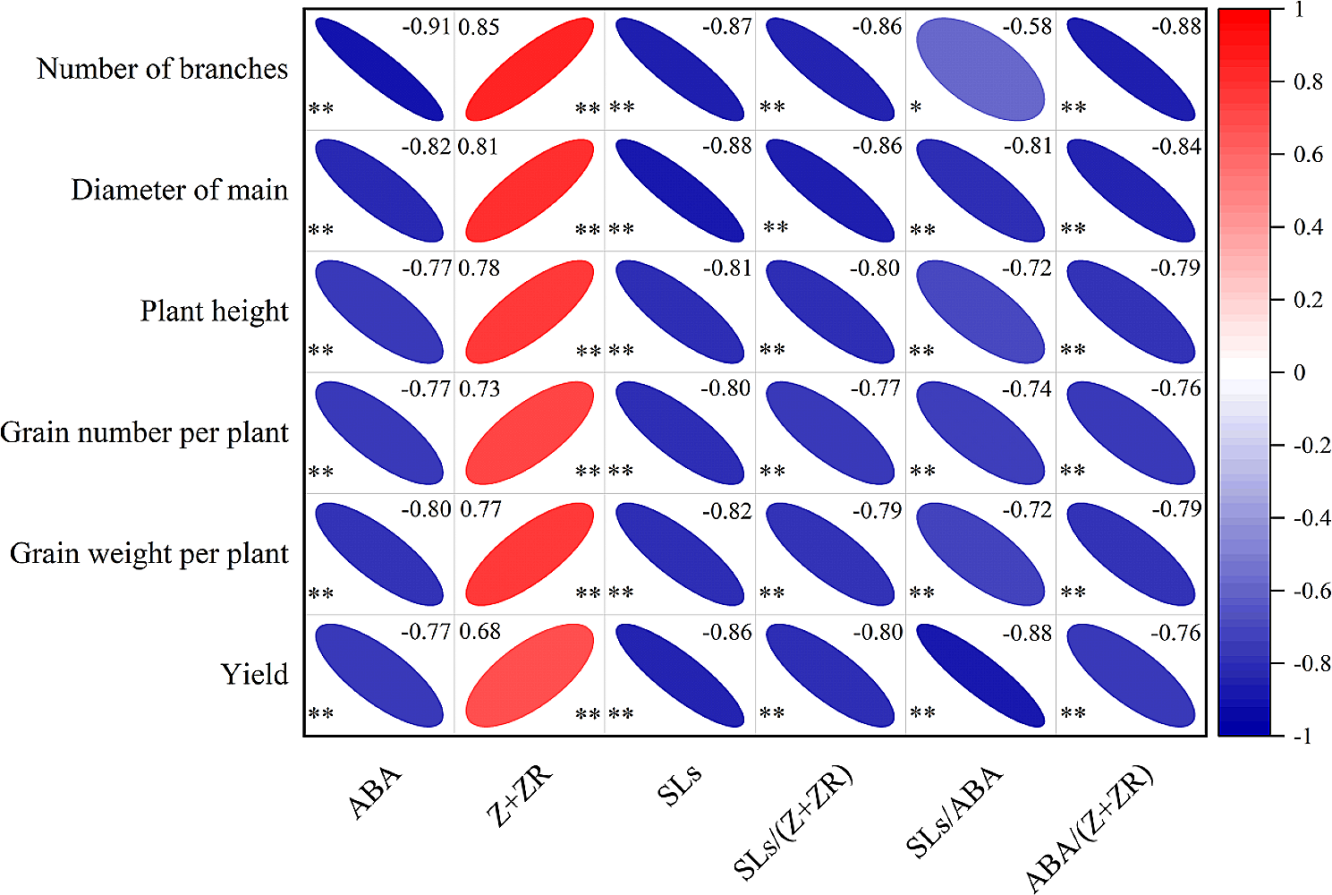
Correlation analysis of hormone levels in plants with buckwheat growth and yield. The numbers in the figure indicate the correlation coefficients between the factors; the significance of the differences between the factors is indicated by * (*P* < 0.05) and ** (*P* < 0.01)

The number of branches, diameter of main stem, plant height, grain number per plant, grain weight per plant, and yield of Tartary buckwheat were significantly negatively correlated with the contents of ABA and SLs, as well as the ratios of SLs/(Z + ZR), SLs/ABA, and ABA /(Z + ZR), whereas they were significantly positively correlated with the content of Z + ZR (Fig. 1). Linear-regression analysis showed that the ratio of ABA/(Z + ZR) can explain 58.4% of the variation in yield, and the ratio of ABA/(Z + ZR) (*p* < 0.001) had a significant negative correlation with yield increase (Table 4).

**Table 4:** Regression analysis between yield and endogenous hormone levels

Regression Equation									
	Non standardized coefficient		Standardization coefficient	t	*P*	VIF	R²	adjust R²	F
	B	standard error	Beta						
constant	3.138	0.274	-	11.456	0.000**	-	0.584	0.552	F = 18.273 *P* = 0.001**
ABA/(Z + ZR)	-0.111	0.026	-0.764	-4.275	0.001**				
y = 3.138-0.111×ABA/(Z + ZR)									

Note: B, indicates the extent of the effect of ABA/(Z + ZR) on yield, with positive values indicating a positive effect and negative values indicating a negative effect; standard error: is B/t value; Standardized coefficients, coefficients obtained by standardizing the data; t: represents the degree of difference between the sample mean and the overall mean, and the larger the absolute value of t, the more significant the difference between the factors is; P, indicates the significance of the difference in the regression equation, ** is *P* < 0.01; VIF, indicates the covariance between factors; R², indicates the degree of fit of the linear regression; F, indicates F test, ** is *P* < 0.01

## Discussion

### Regulation of rac-GR24 and Tis-108 treatment on the growth of Tartary buckwheat

SLs are generally believed to be synthesized in roots and then transported to various parts of the plant [26]. Studies had found that SLs inhibited branching in the branching mutants of pea, rice, and *Arabidopsis* [20, 27]. Su et al. (2020) also found that the branching of rapeseed is inhibited after treatment with rac-GR24 [28]. Some studies suggest that SLs promote the growth of plant stems [29] and plant height [30]. In the present study, compared with the CK treatment, rac-GR24 treatment reduced the number of main-stem branches, whereas the lower concentration of rac-GR24 treatment (G_10_) can promote the growth of main stem and plant height. The application of Tis-108 promoted the number of main-stem branches, main-stem diameter, and plant height. These findings were consistent with the above research results, indicating that rac-GR24 treatment inhibited the branching of Tartary buckwheat and promoted the growth of main stem and plant height of Tartary buckwheat at lower concentrations. This phenomenon may be related to the concentration dependence of SLs on the regulation of plant growth and development [31]. The application of exogenous rac-GR24 may also cause a disorder of IAA transport in Tartary buckwheat, resulting in different changes in hormone levels among cells [21, 32]. Thus, the regulatory effect of SLs on the growth of each part of the main stem of Tartary buckwheat was differentially expressed. The results of this study showed as well that the number of main-stem branches, main-stem diameter, and plant height were inhibited at higher concentrations of rac-GR24 treatment (G_20_). This result was inconsistent with those of Li et al.(2019) [30] and Agusti et al.(2012) [29]. The reason may be related to the different concentrations of rac-GR24 applied and to the differences in tolerance to rac-GR24 among crop varieties.

### Regulation of rac-GR24 and Tis-108 treatment on endogenous hormone levels

By regulating the changes in related hormones in plants, the growth and development of plants can be effectively regulated [33]. As an important hormone regulating plant development and stress resistance, SLs have obvious interaction with CTK and ABA [34]. Due to the same biosynthetic precursors, a synergistic effect exists between ABA and SLs [35]; however, it often shows mutual antagonism with CTK. Duan et al. (2019) found that SLs can stimulate the expression of cytokinin oxidase/dehydrogenase in rice and promote the separation and degradation of CTK, thereby reducing the content of CTK in rice [36]. Yoneyama et al. (2020) believed that the application of exogenous CTK inhibits SL biosynthesis, thereby reducing the production of SLs [24]. In the present study, the application of rac-GR24 significantly increased the content of SLs and ABA in Tartary buckwheat grains and reduced the content of Z + ZR, consistent with the results of Wu et al. (2022) [34] and Duan et al. (2019) [36]. This finding showed that increased SL content promoted the biosynthesis of ABA in Tartary buckwheat [37] and inhibited the accumulation of CTK (Z + ZR) [36]. Tis-108 treatment reduced SLs and ABA content in Tartary buckwheat grains but increased the Z + ZR content, which further confirmed the above view. It indicated that the application of rac-GR24 and Tis-108 can further change the level of SLs in Tartary buckwheat grains, thereby regulating the synthesis of Z + ZR and ABA.

Results of correlation analysis showed that the grain number per plant, grain weight per plant, and yield of Tartary buckwheat were significantly negatively correlated with the contents of ABA and SLs, as well as the ratios of SLs/(Z + ZR), SLs/ABA, and ABA/(Z + ZR), whereas they were significantly positively correlated with the content of Z + ZR. This finding indicated that the application of rac-GR24 changed the hormone levels and balance of SLs, ABA, and Z + ZR in grains, thereby affecting the formation of Tartary buckwheat yield. Differences also existed in the regulatory effects of ABA and Z + ZR on the yield formation of Tartary buckwheat. Increasing the concentration of Z + ZR or reducing the concentration of ABA in the grain can promote grain formation and increase yield, consistent with the results of Abid et al. (2017) [38] and Fu et al. (2013) [39]. This finding may be related to the different regulatory effects of ABA and Z + ZR on grain-embryo development [40]. Results of linear regression also showed that a significant negative correlation existed between the ratio of ABA/(Z + ZR) and the yield of Tartary buckwheat, and it can explain 58.4% of the variation in yield. Thus, rac-GR24 treatment may increase the ABA content and reduce the Z + ZR content in the grain (Table 3), thereby increasing the ratio of ABA/(Z + ZR). The outcome was decreased grain weight, ultimately reducing the yield of Tartary buckwheat.

### Regulation of rac-GR24 and Tis-108 treatment on the yield of Tartary buckwheat

Kelly et al. (2023) found that increased SL content reduces the number of branches and grains, leading to decreased yield of rice and wheat [41]. Some scholars have obtained inconsistent results. Yamada et al. (2019) found that SLs increase yield by regulating endosperm development in rice [19]. Wang et al. (2022) found that the application of exogenous SLs can promote the increase of wheat yield [42]. In the present study, rac-GR24 treatment significantly reduced the number of grains per plant, grain weight per plant, and yield, whereas Tis-108 treatment significantly increased the number of grains per plant, grain weight per plant, and yield of Tartary buckwheat. This result was consistent with that of Kelly et al. (2023) [41] but not with that of Yamada et al. (2019) [19] and Wang et al. (2022) [42]. The reason for the inconsistency may be related to the spatial specificity of Tartary buckwheat grain filling (grain plumpness greatly differ among different parts of Tartary buckwheat) [43]. Another reason may be the rac-GR24-induced reduction in plant height and number of main-stem branches. It decreased the dry matter accumulation, which may also be one of the factors affecting the final yield of Tartary buckwheat.

## Conclusions

The application of exogenous rac-GR24 inhibited branch growth, and the effect of SLs on the growth of main-stem diameter and plant height of Tartary buckwheat changed from promotion to inhibition with increased treatment concentration. This finding indicated that the regulation of SLs on the growth and development of Tartary buckwheat was concentration dependent. Exogenous rac-GR24 affected the grain number and grain weight by regulating the content and balance of endogenous hormones in the grains, which ultimately inhibited the yield of Tartary buckwheat. Therefore, the application of an appropriate concentration of Tis-108 (10µmol/L) can reduce the production of SLs in grains and promote an increase in Tartary buckwheat yield.

### Electronic supplementary material

Below is the link to the electronic supplementary material.

Supplementary Material 1

## Data Availability

The data used and analysed during the current study are available from the corresponding author on reasonable request.
