# Supplementary material for: Strigolactones affect the yield of Tartary buckwheat by regulating endogenous hormone levels
Source: BMC Plant Biol. 2024 Apr 24;24:320. doi: 10.1186/s12870-024-05029-0 (PMC11040829; doi:10.1186/s12870-024-05029-0)
Supplement: Supplementary file 1 — Supplementary Material 1 [file 12870_2024_5029_MOESM1_ESM.docx]

**Supplementary data**

Supplementary data in 2019

**Table S1.** Effects of different concentrations of rac-GR24 and Tis-108 on Agronomic characters and yield of Tartary buckwheat

| Treatment | Number of  branches | Diameter of main  stem (mm) | Plant height  (cm) | Grain number per plant | Grain weight per plant (g) | Yield  (t/ha) |
| --- | --- | --- | --- | --- | --- | --- |
| CK | 14.67±1.15b | 3.00±0.03c | 85.39±1.21c | 258.33±4.04c | 4.58±0.03c | 1.99±0.05bc |
| G_10_ | 11.67b1.15c | 3.18±0.02c | 97.85±0.74b | 221.33±9.71d | 4.11±0.06d | 1.91±0.07c |
| G_20_ | 11.33b1.15c | 2.38±0.14d | 77.04±3.38d | 175.67±7.51e | 3.38±0.22e | 1.51±0.07d |
| T_10_ | 16.00±1.00ab | 3.89±0.06a | 115.19±3.28a | 409.67±12.06a | 8.34±0.20a | 2.37±0.13a |
| T_20_ | 17.33±0.58a | 3.68±0.17b | 114.92±6.14a | 348.67±18.56b | 7.30±0.14b | 2.10±0.03b |

Data are presented as mean ± standard error of the mean. Small letter in the same column means significant difference at *p*<0.05. CK, G_10_, G_20_, T_10_, and T_20_ represent the application of rac-GR24 or Tis108 were 0, 10, and 20μmol/L, respectively.

**Table S2.** Effects of different concentrations of rac-GR24 and Tis-108 on endogenous hormones in Tartary buckwheat grains

| Item | Treatment | Period | | | | |
| --- | --- | --- | --- | --- | --- | --- |
|  |  | 5d | 10d | 15d | 20d | 25d |
| ABA (ng/mL) | CK | 102.81±0.69c | 113.29±0.48c | 116.72±0.53c | 119.16±0.48c | 102.11±0.48c |
|  | G_10_ | 106.26±0.033b | 114.50±0.51b | 122.48±0.40b | 122.29±0.73b | 109.20±0.29b |
|  | G_20_ | 112.65±0.48a | 116.74±0.78a | 126.57±0.19a | 125.36±0.80a | 113.67±0.67a |
|  | T_10_ | 101.60±0.96d | 111.90±0.64d | 109.08±0.97d | 101.35±1.44d | 98.98±0.13d |
|  | T_20_ | 102.69±0.48cd | 101.03±0.73e | 100.07±0.77e | 97.01±0.97e | 93.37±0.73e |
| Z+ZR (ng/mL) | CK | 9.31±0.05a | 10.05±0.04b | 11.77±0.09c | 11.09±0.16b | 10.15±0.21b |
|  | G_10_ | 8.90±0.02b | 9.80±.05c | 10.16±0.03d | 10.35±0.016c | 9.46±0.66c |
|  | G_20_ | 7.95±0.11c | 8.91±0.04d | 10.06±0.08d | 9.44±0.13d | 8.44±0.14d |
|  | T_10_ | 9.38±0.23a | 10.35±0.17a | 14.32±0.07a | 11.67±0.05a | 10.61±0.08b |
|  | T_20_ | 9.52±0.00a | 10.20±0.13ab | 13.02±0.04b | 11.69±0.14a | 11.87±0.12a |
| SLs (ng/mL) | CK | 119.77±0.16b | 135.79±0.70c | 144.91±1.05c | 159.92±.55b | 136.80±0.16c |
|  | G_10_ | 131.19±0.83a | 142.15±1.36b | 177.20±0.16b | 160.38±0.80b | 146.29±0.32b |
|  | G_20_ | 131.65±0.42a | 148.87±0.48a | 188.37±0.28a | 161.39±0.42a | 161.85±0.48a |
|  | T_10_ | 119.03±1.10b | 134.41±0.42d | 135.98±0.58d | 150.77±0.48c | 129.81±1.27d |
|  | T_20_ | 104.02±0.16c | 127.50±0.32e | 136.16±0.28d | 141.50±0.16d | 126.68±1.05e |

Data are presented as mean ± standard error of the mean. Small letter in the same column means significant difference at *p*<0.05. CK, G_10_, G_20_, T_10_, and T_20_ represent the application of rac-GR24 or Tis108 were 0, 10, and 20μmol/L, respectively.

**Supplementary data in 2020**

**Table S3.** Effects of different concentrations of rac-GR24 and Tis-108 on Agronomic characters and yield of Tartary buckwheat

| Treatment | Number of branches | Diameter of main  stem (mm) | Plant height  (cm) | Grain number per plant | Grain weight per plant (g) | Yield  (t/ha) |
| --- | --- | --- | --- | --- | --- | --- |
| CK | 14.00±1.00bc | 2.96±0.04d | 86.45±3.22b | 249.67±6.35c | 4.56±0.06c | 1.99±0.04b |
| G_10_ | 12.33±1.53c | 3.16±0.03c | 96.43±0.71b | 216.67±11.02d | 3.99±0.12d | 1.85±0.09c |
| G_20_ | 12.00±1.00c | 2.58±0.03e | 75.02±3.34c | 184.33±8.08e | 3.14±0.23e | 1.51±0.05d |
| T_10_ | 16.67±1.15a | 3.97±0.10a | 121.17±10.04a | 412.33±27.10a | 8.20±0.35a | 2.43±0.11a |
| T_20_ | 16.00±1.00ab | 3.68±0.08b | 113.16±6.15a | 353.33±16.62b | 7.14±0.08b | 2.06±0.03b |

Data are presented as mean ± standard error of the mean. Small letter in the same column means significant difference at *p*<0.05. CK, G_10_, G_20_, T_10_, and T_20_ represent the application of rac-GR24 or Tis108 were 0, 10, and 20μmol/L, respectively.

**Table S4.** Effects of different concentrations of rac-GR24 and Tis-108 on endogenous hormones in Tartary buckwheat grains

| Item | Treatment | Period | | | | |
| --- | --- | --- | --- | --- | --- | --- |
|  |  | 5d | 10d | 15d | 20d | 25d |
| ABA (ng/ml) | CK | 103.30±0.41c | 113.51±0.20c | 117.08±0.31c | 119.70±0.47c | 102.47±0.31c |
|  | G_10_ | 106.55±0.25b | 115.08±0.50b | 122.90±0.36b | 122.71±0.36b | 109.46±0.22b |
|  | G_20_ | 113.09±0.39a | 117.44±0.61a | 126.76±0.16a | 126.16±0.69a | 114.18±0.44a |
|  | T_10_ | 102.21±0.53d | 111.68±0.18d | 109.78±0.61d | 102.37±0.88d | 99.16±0.16d |
|  | T_20_ | 103.04±0.31c | 101.73±0.61e | 100.77±0.61e | 97.80±0.69e | 93.88±0.44e |
| Z+ZR (ng/ml) | CK | 9.32±0.01b | 10.01±0.04c | 11.72±0.06c | 11.10±0.01c | 10.23±0.07c |
|  | G_10_ | 8.89±0.01c | 9.80±0.01d | 10.20±0.04d | 10.39±0.03d | 9.82±0.31d |
|  | G_20_ | 8.04±0.08d | 8.90±0.02e | 10.13±0.05d | 9.52±0.06e | 8.52±0.06e |
|  | T_10_ | 9.51±0.11a | 10.46±.08a | 14.38±0.05a | 11.62±0.05b | 10.66±0.04b |
|  | T_20_ | 9.50±0.02a | 10.28±0.06b | 13.07±0.03b | 11.71±0.02a | 11.92±0.03a |
| SLs (ng/ml) | CK | 119.77±0.16b | 135.79±0.70c | 144.91±1.05c | 159.92±0.55b | 136.80±0.16c |
|  | G_10_ | 131.19±0.83a | 142.15±1.36b | 177.20±0.16b | 160.38±0.80b | 146.29±0.32b |
|  | G_20_ | 131.65±0.42a | 148.87±0.48a | 188.37±0.28a | 161.39±0.42a | 161.85±0.48a |
|  | T_10_ | 119.03±1.10b | 134.41±0.42d | 135.95±0.58d | 150.77±0.48c | 129.81±1.27d |
|  | T_20_ | 104.02±0.16c | 127.50±0.32e | 136.16±0.28d | 141.50±0.16d | 126.88±1.05e |

Data are presented as mean ± standard error of the mean. Small letter in the same column means significant difference at *p*<0.05. CK, G_10_, G_20_, T_10_, and T_20_ represent the application of rac-GR24 or Tis108 were 0, 10, and 20μmol/L, respectively.
